# The citron homology domain as a scaffold for Rho1 signaling

**DOI:** 10.1073/pnas.2110298118

**Published:** 2021-09-20

**Authors:** Sergio G. Bartual, Wenfan Wei, Yao Zhou, Veronica M. Pravata, Wenxia Fang, Kaizhou Yan, Andrew T. Ferenbach, Deborah E. A. Lockhart, Daan M. F. van Aalten

**Affiliations:** ^a^School of Life Sciences, University of Dundee, Dundee DD1 5EH, United Kingdom;; ^b^College of Life Science and Technology, Guangxi University, Nanning 530004, China;; ^c^State Key Laboratory of Non-Food Biomass and Enzyme Technology, Guangxi Academy of Sciences, Nanning 530007, China;; ^d^Aberdeen Fungal Group, Institute of Medical Sciences, University of Aberdeen, Aberdeen AB25 2ZD, United Kingdom

**Keywords:** *Aspergillus fumigatus*, structural biology, signal transduction, cell wall, citron homology domain

## Abstract

*Aspergillus fumigatus* is a human opportunistic pathogen showing emerging resistance against a limited repertoire of antifungal agents available. The GTPase Rho1 has been identified as an important regulator of the cell wall integrity signaling pathway that regulates the composition of the cell wall, a structure that is unique to fungi and serves as a target for antifungal compounds. Rom2, the guanine nucleotide exchange factor to Rho1, contains a C-terminal citron homology (CNH) domain of unknown function that is found in many other eukaryotic genes. Here, we show that the Rom2 CNH domain interacts directly with Rho1 to modulate β-glucan and chitin synthesis. We report the structure of the Rom2 CNH domain, revealing that it adopts a seven-bladed β-propeller fold containing three unusual loops. A model of the Rho1–Rom2 CNH complex suggests that the Rom2 CNH domain interacts with the Rho1 Switch II motif. This work uncovers the role of the Rom2 CNH domain as a scaffold for Rho1 signaling in fungal cell wall biosynthesis.

## Significance

*Aspergillus fumigatus* gives rise to invasive aspergillosis in immunocompromised individuals. The rise of *A. fumigatus* antifungal resistance threatens a limited arsenal of treatment options. Here, we use genetic and molecular approaches to dissect the contribution of the citron homology (CNH) domain of the guanine nucleotide exchange factor Rom2 in regulating the biosynthesis of the essential and unique fungal cell wall, an important target of antifungal compounds. The CNH domain plays an essential role as a stabilizer for the small GTPase Rho1, a key regulator of glucan biosynthesis. This work provides a model for their interaction, revealing a promising molecular mechanism to explore in the quest for novel antifungal compounds.

Fungal diseases are a major cause of morbidity in immunocompromised patients. *Aspergillus fumigatus*, the causative agent of invasive aspergillosis, is one of the deadliest invasive pathogenic fungi (1). There is a limited repertoire of therapeutic agents available to treat aspergillosis. These include the azoles, which disrupt the synthesis of the fungal membrane by targeting the ergosterol biosynthetic pathway (2, 3); polyenes, which bind directly to ergosterol affecting cell membrane integrity (4, 5); echinocandins, which target cell wall stability by inhibiting glucan synthesis (6, 7); and nucleotide analogs like 5-flucytosine, which hamper fungal DNA synthesis (8). Among them, the azoles voriconazole and isavuconazole are the preferred antifungal treatment for invasive aspergillosis due to their lower toxicity and their higher fungicidal activity (9). However, the emergence of azole-resistant strains provides an impetus for the search of novel targets within pathways that are essential for *A. fumigatus* survival (10).

The fungal cell wall is a highly ordered carbohydrate-rich structure that is not present in human cells but is essential for protecting fungi from challenging environmental factors such as mechanical stress, temperature changes, osmotic stress, pH, and nutrient limitations (11). In addition, the cell wall also mediates host-pathogen interactions that are critical during infection (12). Therefore, the fungal cell wall is considered as a potential source of selective antifungal targets that are unlikely to affect human cells. The 1,3-β-glucan is a major *A. fumigatus* cell wall component that contributes up to 20% to 35% of the cell wall biomass (13). Other components of the *A. fumigatus* cell wall are α-glucan, chitin, galactomannan, and a mixed 1,4-β-/1,3-β-glucan layer (13). The fungal cell wall is highly dynamic with changeable components to counteract different types of stresses. This response is mainly mediated by the cell wall integrity (CWI) signaling pathway in which the Rho-type GTPase homologous 1 (Rho1) protein is a key node (14, 15). The cell membranes of Rho1 mutants in *Saccharomyces cerevisiae*, *Candida albicans*, and *A. fumigatus* were found to be defective in the activity of the plasma membrane 1,3-β-glucan synthase (GS) enzyme, which was rescued by the addition of purified Rho1 (7, 16). Apart from direct binding and subsequent activation of the 1,3-β-GS, Rho1 was also shown to modulate different downstream effectors in budding yeast, including the transcription factor Rlm1 that controls the expression of genes involved in cell wall biosynthesis (14, 15). Furthermore, knockdown of *A. fumigatus rho1* induces lethality through cell lysis (17). Taken together, these studies show that Rho1 activity is critical for fungal survival.

Upstream of Rho1 are the GTP exchange factor proteins (GEFs) Rom1 and Rom2, which, despite their high degree of homology, induce very different phenotypes in knockout experiments. Rom1 is dispensable, whereas the loss of Rom2 induces cell lysis at high temperatures in *S. cerevisiae* (18). In *A. fumigatus*, complete loss of Rom2 (*Af*Rom2) results in a lethal phenotype (19). At the cellular level, Rom2 integrates signals from cell wall stress sensors such as Wsc1-3, Mid1, and Mtl1 to activate Rho1 (14, 15). Furthermore, the subcellular localization of GEFs is directly correlated with their function in different signaling pathways (20). Similar to the reported subcellular localization of the fission yeast Rom2 homolog protein Rgf1p (21), *Af*Rom2 is found at the hyphal tips where it colocalizes with *Af*Rho1 (19). These findings suggest a prominent role of the *Af*Rom2–*Af*Rho1 interaction in fungal cell wall biogenesis.

**Fig. 1.**
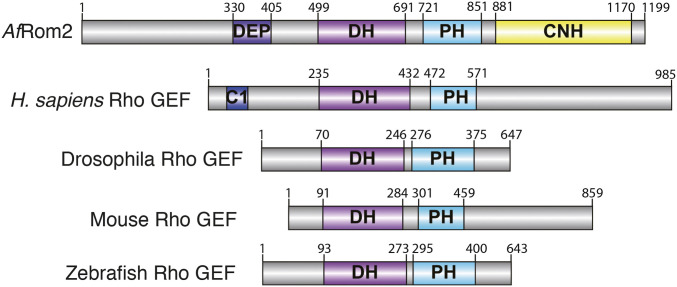
Schematic representation (not to scale) of *Af*Rom2 and comparison with other GEF proteins. Identified domains: DEP domain, phorbol esters/diacylglycerol binding domain (C1), DH domain, PH domain (20), and CNH domain from *A. fumigatus* (*Af*Rom2 Uniprot Q4WUI2) and human, *Drosophila*, mouse, and zebrafish Rho GEF proteins (Uniprot codes: Q92974, Q9VS45, Q5SSL4, and A5 × 7A1, respectively) are annotated in the figure. The residues that define the boundaries of the DEP, DH, PH, and CNH domains are indicated for clarity.

*Af*Rom2 is a multidomain protein (Fig. 1), containing a Dishevelled, Egl-10, and Pleckstrin (DEP) domain that negatively regulates its activity (19), a Dbl Homology (DH) domain that facilitates GDP exchange (20, 22), and a Pleckstrin Homology (PH) domain that interacts with the DH domain and contributes to GEF activity (19, 21, 22). In addition, it possesses a C-terminal citron homology (CNH) domain whose exact function remains unknown, but it has been proposed to interact with either proteins (19, 23–25) or lipids, directing, as a consequence, the cellular location (26).

Here, we use a multidisciplinary approach to uncover the roles of the CNH domain as a driver for the *Af*Rom2 cellular localization and as a scaffold for Rho1 GTPase signaling. We show that the Rom2 CNH domain is critical for *A. fumigatus* cell wall synthesis and stabilizes the 1,3-β-glucan synthesis regulator *Af*Rho1. We report a crystal structure of a eukaryotic CNH domain, revealing that it adopts a seven-bladed β-propeller fold. Together with the crystal structure of *Af*Rho1 in complex with GDP, we identify the putative *Af*Rom2 CNH-*Af*Rho1 binding interface, involving a contribution of the *Af*Rho1 Switch II motif. This work describes the function of *Af*Rom2 CNH as a scaffold for *Af*Rho1 signaling and its essential role in cell wall synthesis.

## Results

### *Af*Rom2 Possesses a C-Terminal CNH Domain of Unknown Function.

Analysis of the *Af*Rom2 (UniProt ID Q4WUI2) primary sequence reveals the presence of the autoinhibitory N-terminal DEP domain (amino acids 330 to 405), the coupled DH and PH domains, sometimes referred simply as GEF domain (19) (amino acids 499 to 851), and a CNH domain (amino acids 881 to 1170) (Fig. 1). In *Drosophila melanogaster*, the CNH domain does not appear to be involved in the cellular localization of Sticky/Citron kinases but is required to interact with Rho GTPase via unknown mechanisms (25). Due to the limited (<20%, *SI Appendix*, Fig. S1*A*) sequence identity between the *Af*Rom2 CNH and the *D. melanogaster* CNH domains, it is unclear if this interaction is conserved in *A. fumigatus*. In contrast, fungal Rom2 CNH domains are highly conserved (*SI Appendix*, Fig. S1*B*) but are absent from Rho GEFs in higher eukaryotes (Fig. 1). In summary, *in silico* analysis reveals that *Af*Rom2 possesses a C-terminal CNH domain conserved in fungi.

### The CNH Domain Contributes to Growth and CWI.

**Fig. 2.**
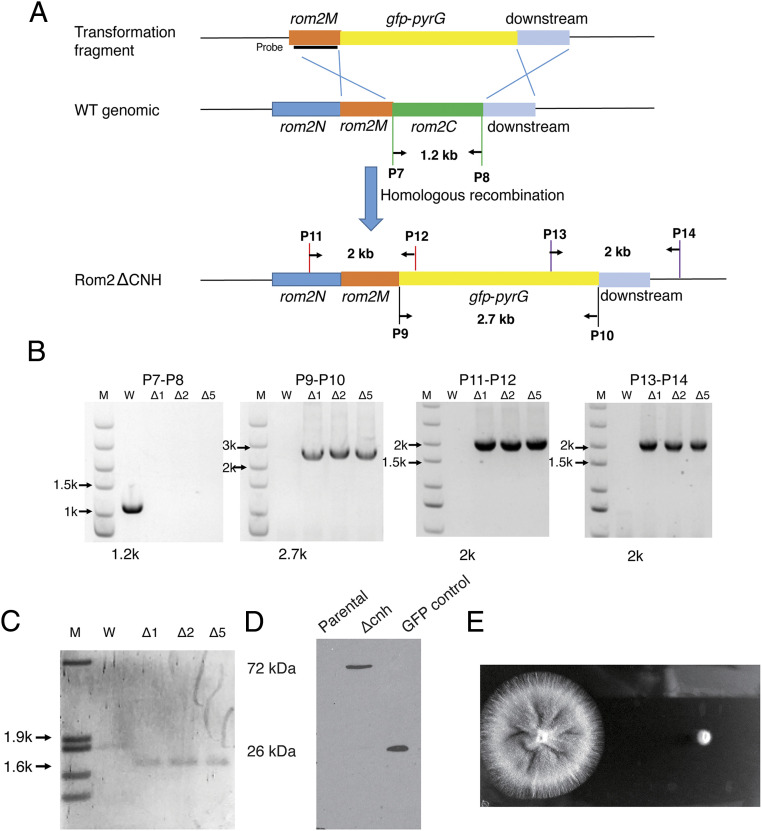
Generation of a *rom2Δcnh* mutant. (*A*) Schematic diagram depicting the strategy for generation of the mutant. (*B*) PCR confirmation of *rom2Δcnh* mutants. Primer pairs of P7-P8 and P9-P10 were used to amplify the *rom2C* and *gfp-pyrG* fragments, respectively. Primer pairs of P11-P12 and P13-P14 were used to amplify regions of −500 bp to +500 bp of homologous arms when correctly integrated into the *rom2C* locus. (*C*) Southern blot confirmation of the *rom2Δcnh* mutants. Genomic DNA was digested by *Xho*I and hybridized using the *rom2M* homologous region as the probe. (*D*) Western blot confirmation using the GFP antibody from cell lysate of the wild-type (WT) and *rom2Δcnh* mutants. Cell lysate of a control strain *gpd-gfp* was used as control. The band size from the *rom2Δcnh* (72 kDa) strain represents the correct molecular weight of *Af*Rom2ΔCNH (44 kDa) with GFP fusion (27 kDa). (*E*) Growth of the *rom2Δcnh* mutant in comparison with the parental strain on solid YEPD medium. The 100 conidia from the *Afrom2Δcnh* mutant and the parental strain were inoculated on YEPD plates and incubated at 37 °C for 48 h.

To investigate the function of the CNH domain in *Af*Rom2, a GFP-tagged *rom2* mutant with the CNH domain replaced by *gfp* and the orotidine 5′-phosphate decarboxylase (*pyr*G) selection marker was constructed and verified by PCR (Fig. 2 *A* and *B*) and Southern blotting (Fig. 2*C*). The resulting mutant strain is hereafter referred to as *rom2Δcnh*. To confirm expression of *Af*Rom2, a GFP antibody was used to detect the truncated GFP-fused *Af*Rom2 in the cell lysate from parental and mutant strains. There was no detectable GFP antibody signal in the parental strain, but a 70 kDa band was detected in the mutant lysate, corresponding to the size of *Af*Rom2ΔCNH (44 kDa) fused with GFP (27 kDa) (Fig. 2*D*).

Conidia of the parental and mutant strains were inoculated onto solid yeast extract peptone dextrose (YEPD) media to explore colony growth phenotypes. After 48 h growth at 37 °C, the colony diameter of the *rom2Δcnh* mutant was reduced when compared to the parental strain, and almost no surface conidia were produced by the mutant (Fig. 2*E*). This finding suggests that the *Af*Rom2 CNH domain is required for normal colony growth and conidia production.

We next generated GFP fusions for the isolated CNH domain and full length *rom2*, integrated into the *A. fumigatus* genome under control of the constitutive *gpd* promoter (27). Constructs were verified by PCR and western blotting (*SI Appendix*, Fig. S3 *B* and *C*). The localization experiments (*SI Appendix*, Fig. S2*A*) show that *Af*Rom2 localizes to the cell membrane, enriched at the hyphal tips where the new cell wall is synthesized, in agreement with a previous study by Dichtl et al. (28). However, in the absence of the CNH domain, *Af*Rom2ΔCNH shows a diffuse distribution in the cytoplasm. Interestingly, the overexpressed, isolated CNH domain localizes to the hyphal tips, suggesting that *Af*Rom2 cellular localization is dependent on its C-terminal CNH domain (*SI Appendix*, Fig. S2*A*).

**Fig. 3.**
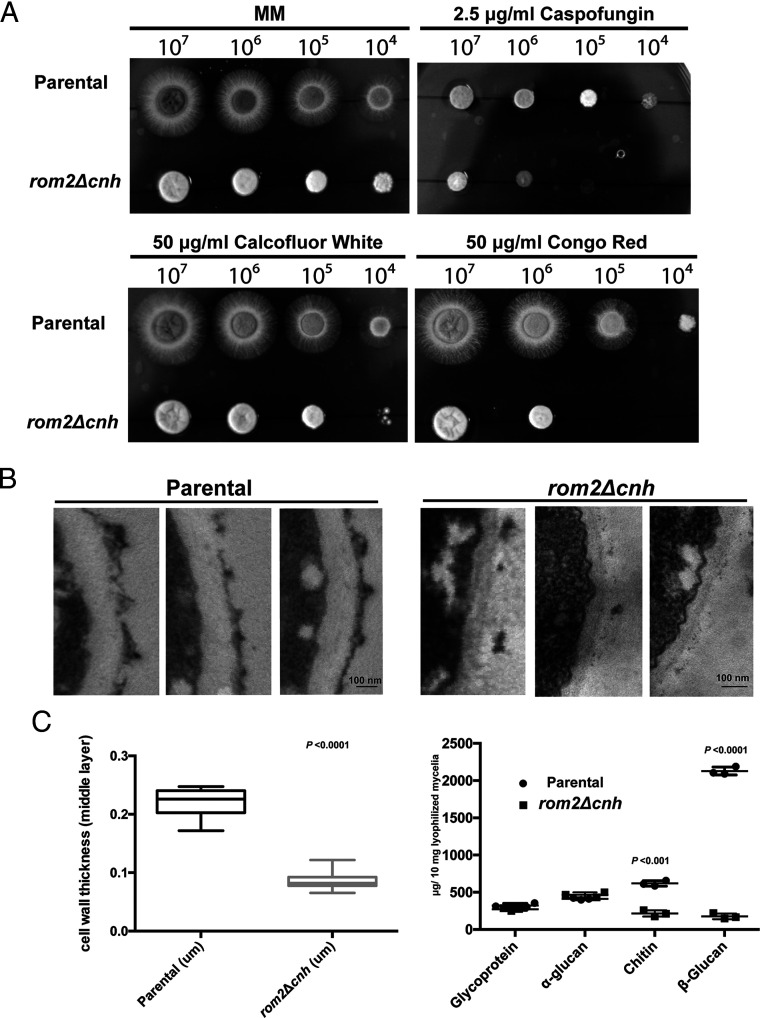
Sensitivity to chemical reagents and cell wall analysis of the *rom2Δcnh* mutant. (*A*) Serial dilutions of conidia from 10^7^ to 10^4^ were spotted onto solid MM containing 50 μg/mL Calcofluor White, 50 μg/mL Congo Red, and 2.5 μg/mL Caspofungin. The colonies were grown at 37 °C for 48 h. The concentrations of the chemicals were chosen as described previously (74). The photographs display representatives of three independent experiments. (*B*) TEM images of representative parental (75) and *rom2Δcnh* mutant hyphal cells. (*C*) Cell wall width measurement of the *rom2Δcnh* mutant compared to the parental strain (*n* > 20) and cell wall composition of the *rom2Δcnh* mutant compared to the parental strain by chemical analysis (56). The plots of the data and the *P* values by the multiple *t* tests were calculated using Prism 6 (GraphPad software). The experiment was performed in triplicate.

We next studied the role of *Af*Rom2 CNH domain in regulating cell wall biogenesis as reported for the full *rom2* gene (19). Serial dilutions of *rom2Δcnh* mutant and parental strain conidia were inoculated on solid minimal media (MM) supplemented with cell wall–perturbing agents (Fig. 3*A*). After 48 h growth at 37 °C, the *rom2Δcnh* mutant showed increased sensitivity to most cell wall–perturbing agents when compared to the parental strain (Fig. 3*A*). Taken together, these data suggest that the CNH domain contributes to growth and CWI.

### The CNH Domain Is Required for Cell Wall Synthesis.

We next investigated whether the sensitivity of the *rom2Δcnh* mutant to cell wall–perturbing agents was the result of a compromised cell wall. Transmission electron microscopy (TEM) was used to examine possible cell wall ultrastructure defects (Fig. 3*B*). Compared to the parental strain, the *rom2Δcnh* mutant shows ∼20-fold reduction in the thickness of the middle region of the cell wall (15 ± 4 nm for the mutant versus 290 ± 30 nm for the parental strain [*n* ≥ 12]) and an associated detachment of the outer layer (Fig. 3*C* and *SI Appendix*, Fig. S2 *B* and *C*).

To investigate the source of this structural defect, we next quantified cell wall carbohydrate components. In agreement with our TEM findings, levels of β-glucan, representing the middle bulk layer of the cell wall, decreased more than 10-fold from 2,100 ± 50 μg/10 mg in the parental strain to only 180 ± 40 μg/10 mg in the *rom2Δcnh* mutant (*P* < 0.0001, Student’s *t* test, *n* = 3, Fig. 3*C*). Furthermore, levels of chitin also decreased from 620 ± 40 μg/10 mg in the parental strain to 220 ± 40 μg/10 mg in the *rom2Δcnh* mutant (*P* < 0.001, Student’s *t* test, *n* = 3, Fig. 3*C*). Glycoprotein and α-glucan levels were unaffected (Fig. 3*C*). These data suggest that the *Af*Rom2 CNH domain is required for cell wall synthesis, affecting levels of the key fungal cell wall components β-glucan and chitin.

### The CNH Domain Interacts with AfRho1.

To identify the mechanism underpinning the role of the *Af*Rom2 CNH domain in regulating cell wall synthesis, we employed a pull-down assay to identify potential CNH binding partners using the GFP-tagged *cnh* construct integrated into the *A. fumigatus* genome (*SI Appendix*, Fig. S3*A*). A *gfp* construct integrated into the genome under control of the same *gpd* promoter was used as a negative control (*SI Appendix*, Fig. S3*D*). We identified 1,236 proteins that uniquely bind GFP-CNH domain compared to the GFP control strain (*SI Appendix*, Fig. S3*E* and Dataset S1). Gene Ontology (GO) enrichment analyses identified that the CNH mainly binds membrane (GO ID: 16020, 27.6%) and plasma membrane (GO ID: 5886, 10.5%) proteins, which is in line with the reported cellular localization of Rom2 and our localization experiments. Of note, we observed cytoskeletal proteins (GO ID: 5856, 3.9%) and several proteins involved in actin regulation, such as End3, the actin-related protein 4, and Pan1. Additionally, 37 of the detected proteins are components of the cell wall, supporting the phenotype seen in the *rom2Δcnh* mutant (Dataset S1). Among these, we identified key proteins belonging to the CWI signaling pathway, including Rho1, MidA, and Fks1.

Given that Rho1 has a key role in regulating glucan synthesis (29), we next used biolayer interferometry (BLI) to further investigate the interaction between *Af*Rho1 and *Af*Rom2 CNH. We used recombinant *Af*Rom2 CNH as the stationary phase to measure affinity toward recombinant *Af*Rho1 in the presence or absence of GDP or the nonhydrolysable GTP analog GTPγS. These experiments revealed dose-dependent binding to *Af*Rom2 CNH with *K*_d_s in the midmicromolar range (*SI Appendix*, Fig. S4). Taken together, these data suggest a direct interaction between the *Af*Rom2 CNH domain and *Af*Rho1.

### The CNH Domain Possesses a Unique β-Propeller Fold.

There is currently no structure of any CNH domain available to aid in generating hypotheses on the nature of the *Af*Rom2 CNH-*Af*Rho1 interaction. We used X-ray crystallography to determine the protein structure of the recombinantly produced *Af*Rom2 CNH domain (residues 862 to 1194) using a selenomethionine phasing strategy. Crystals belonging to the orthorhombic space group *P*2_1_2_1_2 with a single molecule in the asymmetric unit diffracted to a maximum of 2.0 Å. The structure was refined to the maximum available resolution with *R* and *R*_*free*_ values of 20.7% and 24.7%, respectively. Data collection and refinement parameters are listed in Table 1.

**Table 1.** Data collection and refinement statistics

	SeMet *Af*Rom2CNH	*Af*Rom2CNH_native	*Af*Rho1-GDP	*Af*Rho1-GTPγS
Space group	*P 2*_*1*_ *2*_*1*_ *2*	*P 2*_*1*_ *2*_*1*_ *2*	*P 4*_*3*_	P 2_1_ 2_1_ 2
*a*, *b*, *c* (Å)	77.0, 92.3, 52.3	78.0, 93.7, 52.6	53.8, 53.8, 62.7	37.2, 65.8, 75.0
Wavelength (Å)	0.97961	0.92818	0.98854	1.54178
T (K)	100	100	100	100
Resolution range (Å)	59.1–2.4	45.9–2.0	100.0–1.4	30.1–2.5
Unique reflections	15,324 (1,415)	174,698 (13,083)	140,126 (7,474)	6,797 (751)
Mean *I*/σ(*I*)	17.5 (2.3)	17.4 (1.3)	18.8 (1.6)	6.3 (3.9)
Multiplicity	2.0 (2.0)	4.0 (4.0)	4.1 (2.2)	6.6 (6.6)
Completeness (%)	100 (100)	99.9 (100)	99.0 (96.8)	100 (100)
R_-merge_	0.04 (0.30)	0.02 (0.57)	0.04 (0.34)	0.18 (0.35)
R_-meas_	0.06 (0.42)	0.03 (0.81)	0.06 (0.48)	0.21 (0.41)
Refinement				
* R*_work_/*R*_free_*^,^†	0.31/0.36	0.22/0.26	0.15/0.18	0.20/0.26
Number of nonhydrogen atoms		2,526	1,438	1,606
Macromolecules		2,434	1,404	1,414
Ligands		0	34	33
Solvent		111	211	159
Protein residues		310	181	182
*B*-factor (Å^2^)				
Macromolecules		56.45	24.81	19.33
Ligands		42.07	19.67	10.60
Solvent		52.70	25.64	22.05
rms deviations				
rms (bonds)		0.017	0.018	0.016
rms (angles)		2.01	2.04	1.83
PDB code		5O51	5ZVP	6JIK

Value for the highest resolution shell is shown in parentheses.

* *R*_*work*_ = Σ_h_||F_obs_| − |F_ca|c_||/Σ_h_|F_obs_|, where F_calc_ and F_obs_ are the observed and calculated structure factors for the reflection *h*.

^†^ *R*_*free*_ = is equivalent to *R*_*work*_ calculated with 5% of flagged reflections not used in refinement.

**Fig. 4.**
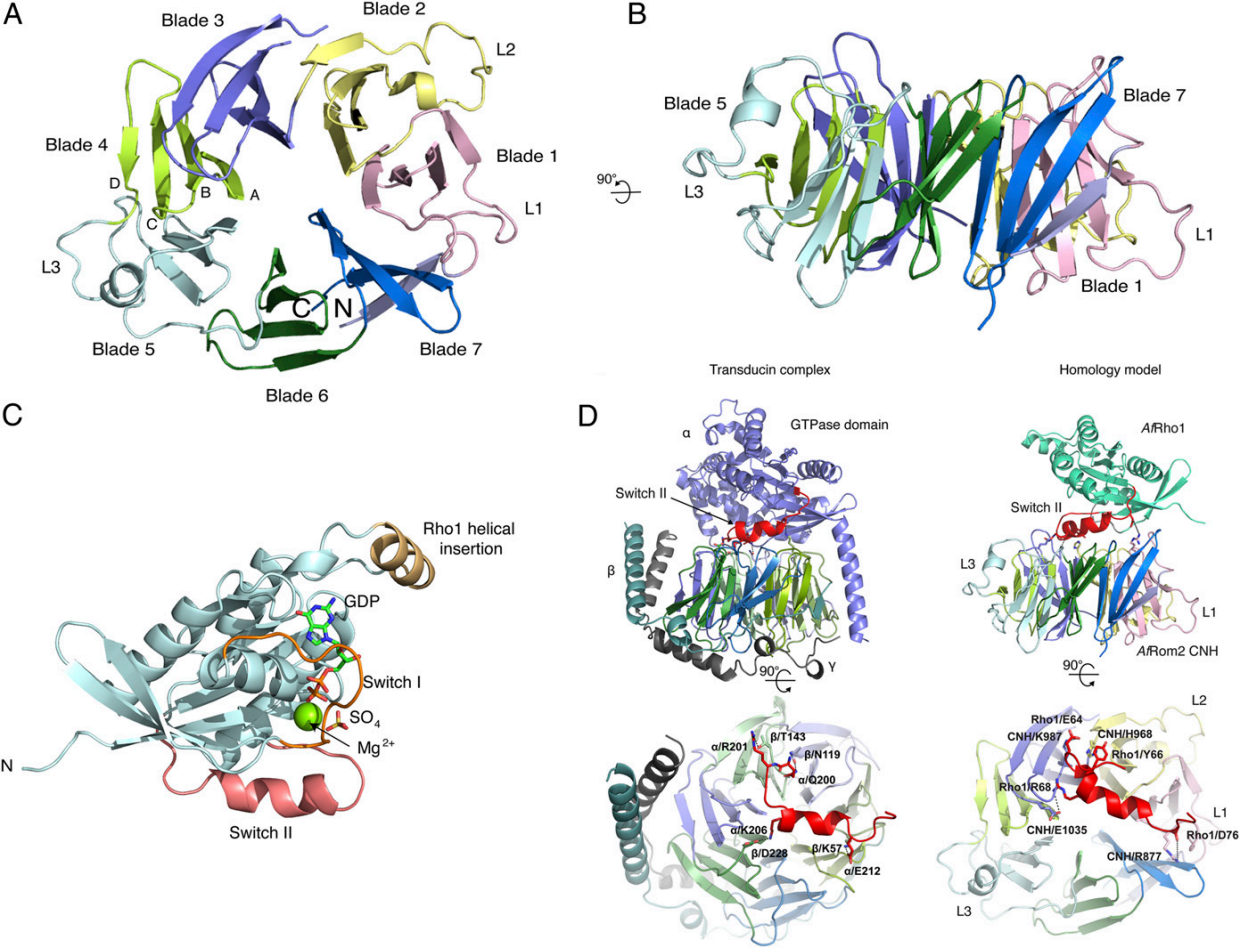
Crystal structures of *Af*Rom2 CNH, *Af*Rho1, and a model of their complex. (*A*) Cartoon representation of the *Af*Rom2 CNH domain structure. Blades and strands are labeled according to the nomenclature commonly used for the β-propeller proteins. Each blade has been colored differently for clarity. The additional CNH domain loops L1, L2, and L3 are indicated. (*B*) 90 degrees rotation of the CNH domain. (*C*) Cartoon representation of the *Af*Rho1 structure in complex with GDP. Nucleotides and magnesium are shown as sticks and spheres, respectively. (*D*) Model of *Af*Rho1–*Af*Rom2 CNH domain complex produced by superposition of *Af*Rho1 and *Af*Rom2 CNH onto α and β transducin, respectively. The transducin heterotrimer (PDB 1GOT) is shown for comparison. *Af*Rom2 CNH blade coloring follows the same pattern as in *A*. The *Af*Rho1 Switch II helix is colored red. The main predicted protein–protein interactions discussed in the main text are labeled.

The *Af*Rom2 CNH domain structure adopts a canonical β-propeller fold containing seven blades, each composed of four antiparallel strands (30). The seven blades are connected by small loops and arranged in a circular fashion, with the last blade formed by the N-terminal and C-terminal strands providing additional stability through an extra zipper of hydrogen bonds (Fig. 4*A*). A structural homology search with the Dali server (31) identified the bacterial β-propeller protein YncE (rmsd = 2.8 Å on 273 Cα atoms, Z value = 21.2) and the apoptotic protease activating factor 1 (rmsd = 3.1 Å on 271 Cα atoms, Z value = 20.7) to be the closest *Af*Rom2 CNH structural homologs (*SI Appendix*, Fig. S5*A*), albeit with poor conservation of sequence (12% and 8% sequence identity, respectively). This discrepancy between sequence and structural homology is common for β-propeller folds (32). Interestingly, the *Af*Rom2 CNH blades are stapled together by a unique complex network of leucine and isoleucine hydrophobic interactions, which deviates from the main β-propeller stapling motifs, such as the WD40 motif, the KELCH motif, or the YWTD motif (30) (*SI Appendix*, Fig. S5*B*). The insertion of a disordered loop between blades 1 and 2 (named L1) and two long loops containing a short helical domain between the strands C and D from blades 2 and 5 (named L2 and L3, respectively) changes the overall shape of the *Af*Rom2 CNH domain to be oval rather than circular (Fig. 4 *A* and *B* and *SI Appendix*, Fig. S5*A*). This results in a cross-propeller diameter 1.5 times larger than the diameter along the propeller axis. Similar to the structural homolog YncE, the presence of the α-helical insertions may confer specific functions (33). Interestingly, sequence alignments suggest that these insertions are also present in other CNH domain proteins, including the *Drosophila* GEFs and *H. sapiens* citron kinases (*SI Appendix*, Fig. S5*A*). Taken together, the structural data show that the CNH domain possesses a unique β-propeller fold.

### The Switch II Region Is a Potential Interface in the AfRho1–AfRom2 CNH Interaction.

To shed light on the observed *Af*Rom2 CNH-*Af*Rho1 interaction, we attempted to obtain crystals of the *Af*Rom2 CNH-*Af*Rho1 complex, but this was not successful. To aid in subsequent modeling experiments, we determined the structure of *Af*Rho1 and obtained crystals in the presence of GDP or GTPγS. Initial phases were determined using the structure of the human GTPase RhoA (*Hs*RhoA, PDB ID: 1FTN, 75% sequence identity with *Af*Rho1) as a phase donor in a molecular replacement experiment (34). Data collection and refinement statistics are shown in Table 1. The overall structure of *Af*Rho1 has a typical Rho GTPase fold, containing a core β-sheet flanked by α-helices (Fig. 4*C*). GDP, GTPγS, and the magnesium cofactor were fully defined by unbiased *F*_*o*_*-F*_*c*_ electron density maps (*SI Appendix*, Fig. S6*A*). In both structures, the nucleotide sits in a positively charged conserved pocket containing Lys18, Asp120, and Arg162, which form interactions with the phosphate groups (*SI Appendix*, Fig. S6*A*).

Similar to the structure of *Hs*RhoA (rmsd = 1.2 Å on 177 Cα atoms; *SI Appendix*, Fig. S6*B*) (34), *Af*Rho1 contains a Switch I region predicted to bind to the GEF domain, a helical Switch II region reported to participate in protein–protein complexes with specific GTPase activators (35), and the canonical Rho GTPase three-turn insertion helix (residues 124 to 134) involved in interactions with, and subsequent activation of, downstream proteins (36) (Fig. 4*C* and *SI Appendix*, Fig. S6*C*). Nucleotide-induced conformational changes in the switch I/II regions of GTPases are known to modulate the interactions with their effectors. Although superposition of the *Af*Rho1 GTPγS/GDP complexes gives an overall rmsd fit of 0.8 Å over 150 aligned Cα atoms, the Switch I and Switch II regions undergo conformational changes (maximum Cα shifts of 8 Å with 47° rotation and 7 Å with 70° rotation, respectively; Video S1).

In absence of an experimental *Af*Rho1–*Af*Rom2 CNH complex, we took advantage of structural similarities of the individual *Af*Rho1 and *Af*Rom2 CNH structures to components of the canonical G-heterotrimeric transducin complex structure, a master regulator of the G protein coupled receptors known to bind and regulate Rho guanine nucleotide exchange factors (Protein Data Bank [PDB]: 1GOT) (35). We superposed the *Af*Rom2 CNH domain onto β-transducin (rmsd = 3.2 Å on 338 Cα atoms) and *Af*Rho1 onto α-transducin (rmsd = 2.6 Å on 177 Cα atoms) (Fig. 4*D*). In this model, the *Af*Rho1 α-helical Switch II motif interacts with *Af*Rom2 CNH similarly to the interaction in the G-heterotrimeric transducin complex between the Gα GTPase and the Gβ β-propeller subunits that facilitates the activation of the signaling G protein by GDP to GTP exchange in the Gα subunit (35). The electrostatic interaction that bridges the Gα Switch II region to the β-propeller domain (Gα Glu212 to Gβ Lys57) is conserved in our homology model (Asp76 of *Af*Rho1 to Arg877 of *Af*Rom2 CNH) (Fig. 4*D*). Additional interactions found in the G-heterotrimeric complex, such as the hydrogen bonds between β-transducin Asn119 and α-transducin Gln200 or β-transducin Thr143 to α-transducin Arg201, are mimicked in the model of the complex by the pairs *Af*Rom2 CNH His968 to *Af*Rho1 Tyr66 and *Af*Rom2 CNH Lys987 to *Af*Rho1 Glu64, respectively (Fig. 4*D*).

Interestingly, *Af*Rho1 lacks the long Gα N-terminal helix that interacts with the lateral face of the Gβ propeller. In the model of the *Af*Rho1–*Af*Rom2 CNH complex, the equivalent interacting interface is occupied by the unique *Af*Rom2 CNH L1 loop (Fig. 4*D* and *SI Appendix*, Fig. S7). Therefore, in our model, the presence of the L1 loop together with the lack of an additional N terminus helix in *Af*Rho1 restricts the *Af*Rho1–*Af*Rom2 CNH complex interface to the flat surface of the CNH and the *Af*Rho1 Switch II region. Taken together, these analyses suggest that the Switch II region is a potential interacting element in the *Af*Rho1–*Af*Rom2 CNH complex.

## Discussion

The CNH domain is a C-terminal motif of unknown function present in fungal Rom2 GEF proteins that are essential for cell wall biogenesis (19, 37, 38). CNH domains are also present in the citron kinases from which they take their name (39), sharing less than 20% sequence identity with the fungal CNH domains, but are absent from the human Rom2 GEF orthologs. Therefore, due to the low sequence identity between fungal and human CNH domains, it is a potential target for the development of selective antifungal compounds. To test this hypothesis, we used the opportunistic fungus *A. fumigatus* deletion mutant *rom2Δcnh* as a model to dissect the function of the fungal CNH domain. This mutant has a severe cell wall defective phenotype that correlates with the previously reported cell wall defects for the conditional *rom2* knockdown mutants (19, 37). Phenotypes of our *rom2Δcnh* strain include cytoplasm leakage and cell lysis, two observations previously reported for the budding yeast conditional Rho1 mutant (17). *Af*Rom2 localizes to the cell membrane in the hyphal tips where the new cell wall is synthesized and is known to interact with the critical cell wall regulator *Af*Rho1 (19, 28). In agreement with Dichtl et al. (28), our data indicate that the localization of *Af*Rom2 in the membranes is driven by the CNH domain, whereas deletion of the CNH domain induces Rom2 membrane dissociation. Thus, the lack of the CNH domain may negatively affect the Rom2–Rho1 interaction. The complete inactivation of Rho1 in budding yeast or Rom2 in *Aspergillus* induces lethality (17, 19). While the *rom2Δcnh* strain shows retarded growth, lethality is only achieved in the presence of cell wall–perturbing agents (Fig. 3*A*). This observation, which is similar to the reported conditional *Afrom2* knockdown phenotype (19), suggests a link between the CNH domain, Rho1, and the CWI signaling pathway, which needs both proteins to maintain CWI under stress conditions.

Upon activation by Rom2 GEF, Rho1 participates primarily in the regulation of the fungal cell wall composition in response to stress by directly modulating the activity of the membrane-bound 1,3-β-GS complex (40). In line with this, analysis of our *rom2Δcnh* mutant cell wall ultrastructure by electron microscopy (EM) revealed a 20-fold reduction thickness of the cell wall middle layer due to a reduction in β-glucan (Fig. 3*B*). This reduction in 1,3-β-glucan may be a consequence of reduced GS activity due to the inability of the *Af*Rom2ΔCNH protein to colocalize in the membranes and activate enough Rho1. This observation correlates with the previously reported *S. cerevisiae* conditional Rho1 knockout strains (7, 16). We therefore propose that the *Af*Rom2 CNH domain is essential for the production of 1,3-β-glucan via Rho1 activation.

Furthermore, in our pull-down experiment using the isolated CNH domain as bait, we identified several components of the CWI signaling pathway reported to interact with Rom2, such as Fks1, MidA, and Rho1 (19). The direct interaction of the isolated *Af*Rom2 CNH domain with *Af*Rho1 under saturating nucleotide concentrations was determined to be in the midmicromolar range (K_d_ = 200 μM), up to three times weaker than the weakest reported GEF-GTPase interaction [range of K_d_ = 0.2 to 77 μM (41)]. In absence of nucleotide, Rho1 interacts with the isolated *Af*Rom2 CNH domain with an affinity of K_d_ = 80 μM. Taken together, we propose that the *Af*Rom2 CNH domain functions as an interactor of *Af*Rho1, acting as a critical element in cell wall biogenesis mediated by the CWI pathway. In addition to the classical CWI pathway, *S. cerevisiae* Rho1 is known to participate in remodeling of the cell wall by regulating actin cytoskeleton polarization (42). It is worth noting that several proteins involved in actin regulation, such as End3, the actin-related protein 4, and Pan1, are detected among the CNH-interacting proteins. It is possible that in addition to its role in the CWI pathway, the CNH domain may affect actin polarization or, as was proposed by Dichtl et al. (17), the actin regulation proteins may interact with Rho1 and thus coelute in our pull-downs.

To understand the molecular mechanisms underpinning the *Af*Rho1–*Af*Rom2 CNH interaction, we first tried to determine the crystal structure of the complex of *Af*Rho1 with the CNH domain of *Af*Rom2. This was unsuccessful, possibly as a result of the relatively weak (midmicromolar) interaction or the destabilization by the high salt conditions used in crystallization screens (43). We also attempted cryo-electron microscopy, but the small size of the *Af*Rho1*–Af*Rom2 CNH complex precluded identification of suitable particles in EM grids. In addition, although we had direct evidence of the Rho1–Rom2 interaction, our BLI experiment does not show a difference in binding between the active Rho1 (GTP complexed) and the relaxed Rho1 (GDP complexed), which, in agreement with the data published for the *Drosophila* Rom2 CNH ortholog Sticky (25), indicates that interaction with the CNH domain does not depend on the Rho1 conformational state and might instead play a scaffolding role in the Rom2–Rho1 interaction.

To shed light on the nature of the interaction, we first obtained the *Af*Rom2 CNH crystal structure, revealing that it possesses a seven-bladed β-propeller fold. Many β-propeller proteins are known to have a flat surface that is used to establish electrostatic interactions with their interactors (44, 45). In pull-down experiments, the isolated *Af*Rom2 CNH domain appears to recruit a large number of proteins that, in absence of further validation, may not necessarily act as *Af*Rom2 interactors. Despite this observation, the CNH structure is remarkably similar to the extensively characterized heterotrimeric G protein Gβ subunit that binds the Gα GTPase subunit in the presence of GDP, which allowed us to predict the putative CNH interaction surface. In the proposed model of the *Af*Rho1–*Af*Rom2 CNH interaction, equivalent stabilizing interactions are mediated by the *Af*Rho1 Switch II motif (46). Interestingly, similar to the findings reported for the *Drosophila* Rom2 CNH ortholog Sticky, the observed interactions are not affected by the switch II conformational changes upon nucleotide exchange (25). The lack of additional anchoring points, although unexpected, has previously been observed in protein–protein interactions for other β-propeller proteins. For example, the non-GTPase protein Laminin establishes a complex with the six-bladed β-propeller Nidogen (PDB 1NPE) that is only stabilized by the packing of a Laminin Switch II–like loop with the surface of the Nidogen β-propeller blades 2 and 3 (*SI Appendix*, Fig. S7) (47). Taken together, the model suggests that the *Af*Rho1 Switch II region is the primary mediator of the *Af*Rom2 CNH–*Af*Rho1 interaction.

The multilayered cell wall is a structure unique to fungi and absent in human cells. Targeting fungal cell wall biosynthetic pathways is thought to be a promising strategy for the development of new antifungal agents. In this work, we have identified a critical stabilizing motif conserved in all fungi, the CNH domain, essential for the molecular crosstalk between Rho1 and Rom2 as part of the CWI pathway. The data presented here show that the presence of the Rom2 CNH domain is required for proper Rho1 localization. Deletion of the Rom2 CNH domain has severe consequences for the CWI signaling pathway, including the disruption of the CWI, leading to cell lysis under stress conditions. Both *Af*Rho1 and *Af*Rom2 are potential antifungal targets, as their knockouts induce lethal phenotypes (17, 19). However, *Af*Rho1 is almost identical to its human ortholog *Hs*RhoA, with both proteins sharing a nearly fully conserved active site (34). *Hs*RhoA is ubiquitous and mediates the cytoskeletal response to external signals in human cells (34). Deficiencies in *Hs*RhoA are not compatible with life (48). Thus, selectively targeting *Af*Rho1 may be difficult and prone to adverse consequences due to collateral inhibition of *Hs*RhoA. However, due to the low degree of conservation between fungal and human CNH domains, targeting this domain or the CNH-Rho1 Switch II binding interface could be a strategy to disrupt CWI signaling. Future work will be needed to explore whether the CNH-Rho1 interface can be specifically targeted.

## Materials and Methods

### Strains and Growth Conditions.

The *A. fumigatus* strain *KU80Δ* was used for phenotypic analysis (49). The parental strain for genetic manipulation, *KU80ΔpyrG^−^* (49), was propagated at 37 °C on solid MM (50) supplemented with 5 mM uridine and 5 mM uracil. Conidia were prepared by propagating strains on solid medium for 72 h at 37 °C. The spores were collected with 0.1% (vol/vol) Tween 20 in physiological saline, washed twice, and resuspended in sterile water. Conidial concentration was confirmed using a hemocytometer and viable cell counting. Mycelium were obtained by inoculating strains in liquid medium and incubating at 37 °C with shaking at 200 rpm. At a specified timepoint, the mycelia were harvested, washed with sterile water, frozen in liquid nitrogen, and ground up using a mortar and pestle. All plasmids were propagated in *Escherichia coli* DH5α cells (Bethesda Research Laboratories).

### Construction of the *rom2Δcnh* Mutant.

Plasmid 434 (51) was obtained from the Fungal Genetics Stock Center (FGSC) and used as an *Afrom2* gene carrier backbone in the construction the *rom2 cnh* deletion mutant. The *gfp* and *pyrG* selection markers were amplified from plasmid pHL83 (52) (obtained from FGSC) with primers P1, containing a *Xba*I restriction site, and P2, containing a *Spe*I restriction site (Table 2). The *rom2 cnh* domain 5′ flanking region (1674-2724) and a noncoding region of *rom2* (1,000 bp) were amplified from plasmid 434 with primers P3 and P4 and primers P5 and P6, respectively (Table 2). The PCR product of *cnh* 5′ flanking region (1,050 bp) and the *rom2* downstream noncoding region were cloned by a restrictionless method (53) into the upstream and downstream regions of the plasmid 434 fusion cassette to create plasmid *rom2* (1674-2724) *gfp*-*pyrG+* (Fig. 2*A*).

**Table 2.** PCR primers used in this study

Primer	Sequence (5′ to 3′)
P1	5′ AAA​TCT​AGA​ATG​AAC​AAG​ACA​GTT​TTG​TGT​TCA​ATT​TTT​TC 3′
P2	5′ CTG​TCT​GAG​AGG​AGG​CAC​TGA​TG 3′
P3	5′ CAT​CAC​CGA​ATT​CTG​GCA​ATG​TCT​AGA​GTG​TTC​GGA​AAT​TGC​TTG​GAA​GTT​CTG 3′
P4	5′ GCT​CCA​GCG​CCT​GCA​CCA​GCT​CCA​ACA​GGG​ACA​AGG​CAG​TTC​ACG​C 3′
P5	5′ CGC​ATC​AGT​GCC​TCC​TCT​CAG​ACA​GGG​CGC​TTG​AAT​GGC​TGG​TAC​AAT​CAA​AAG 3′
P6	5′ CGG​AGA​GAG​ATT​CTT​CTG​CTG​CTG​TAC​TAG​TAT​GGT​ATC​TGG​CTG​TTG​CTT​GCT​CCC​G 3′
P7	5′ CAC​TCC​CAA​TAC​TAT​CCT​CTT​GCG​C 3′
P8	5′ GGC​TTG​TTC​CAG​AAA​TCC​AGA​CTG​GC 3′
P9	5′ GGA​GCT​GGT​GCA​GGC​GCT​GGA​GC 3′
P10	5′ CTG​TCT​GAG​AGG​AGG​CAC​TGA​TGC​G 3′
P11	5′ CGA​TGT​GAC​GTA​CGA​TCA​TCG​ACT​TC 3′
P12	5′ CTT​GTC​GGC​CAT​GAT​GTA​TAC​GTT​GTG 3′
P13	5′ GGA​CAG​CAA​TAC​CAG​ACT​CCT​GCA​TC 3′
P14	5′ GCA​GTG​GTC​ACG​ATT​CCT​CTA​TGA​AC 3′
P15	5′ CAT​CAC​CGA​ATT​CTG​GCA​ATG​TCT​AGA​atg​AGT​AAA​GGA​GAA​GAA​CTT​TTC​ACT​GG 3′
P16	5′ GAG​CAT​TGT​TTG​AGG​CGA​CCG​GTT​TAC​TTG​TTC​CAG​AAA​TCC​AGA​CTG​GC 3′
P17	5′ CGC​CAA​GGC​TTT​ATC​TAT​GTA​G 3′
P18	5′ TCC​CAT​GAG​ATC​TTC​CAA​TCC​G 3′
P19	5′ CAG​TCT​GGA​TTT​CTG​GAA​CAA​GTA​ACA​ACA​ACA​ACA​ATG​AGC​GGC​C 3′
P20	5′ GGC​CGC​TCA​TTG​TTG​TTG​TTG​TTA​CTT​GTT​CCA​GAA​ATC​CAG​ACT​G 3′
P21	5′ CTG​GGA​TCC​ATG​GCT​GAA​TCC​GCC​GCA​AGC 3′
P22	5′ GAT​GCG​GCC​GCT​CAT​CAG​TGG​GTC​TTG​GTC​AAG​AGA​GCA​G 3′
P23	5′ CTG​GGA​TCC​AAC​AAG​ACA​GTT​TTG​TGT​TCA​AAT​TTT​TTC 3′
P24	5′ GAT​GCG​GCC​GCT​CAT​TGT​TGT​TGT​TGG​GGC​TTG​TTC​C 3′

Following DNA sequence verification, *rom2(1674-2724)-gfp-pyrG+* was transformed into *A. fumigatus KU80ΔpyrG^−^* by polyethylen glycol (PEG)-mediated fusion of protoplasts (54). Positive transformants were selected by uridine/uracil autotrophy and verified by PCR and western blot analysis. For PCR confirmation, a pair of primers P7 and P8 were used to amplify the *cnh* region (*rom2C*) of the *rom2* gene. The product was obtained from the parental strain but not from the mutant, suggesting the successful deletion of the *cnh* coding region in the *rom2* gene. Primers P9 and P10 were used to amplify the *gfp-pyrG* cassette from the mutant but not from the parental strain, demonstrating that the *gfp-pyrG* cassette has been incorporated into the genomic DNA of the mutant. Primers P11 and P12 were used to amplify a region from the *rom2* gene (1.4 kb upstream of *cnh*) to the 5′ region of the *gfp-pyrG* cassette (493 bp at the 5′ region). The product was only obtained from the mutant but not from the parental strain. Furthermore, primers P13 and P14 were used to amplify the region from 3′ of the *gfp-pyrG* (526 bp) cassette to the downstream 1.5 kb of *cnh*. Again, the product was only obtained from the mutant but not from the parental strain. Primer pairs of P11-P12 and P13-P14 were used to demonstrate that the *gfp-pyrG* cassette has been incorporated into the correct locus in the *A. fumigatus* genome. To further validate the mutant, Southern blotting was conducted. Using the left homologous region as probe and *Nco*I digestion of genomic DNA, the hybridization band of the mutant (1,628 bp) was expected and different from the band of the parental strain (1,853 bp), demonstrating the single correct integration of the deletion cassette at the CNH domain locus.

Conidia from the mutant and the parental strain (negative control) were inoculated in 10 mL of YEPD (2% yeast extract, 2% glucose, and 0.1% peptone) and cultured in a flask rotator at 37 °C for 48 h. Cell extracts were prepared by homogenizing the mycelia using liquid nitrogen in lysis buffer (50 mM Tris/HCl pH 7.5, 150 mM NaCl, 50 mM KCl, 0.01% Triton X-100, 1 mM phenylmethylsulfonyl fluoride (PMSF), and 1:100 protease inhibitor mixture). Cell lysates were centrifuged at 5,000 rpm for 30 min to eliminate cell debris, and then supernatants were further centrifuged at 7,000 rpm for 10 min at 4 °C. Total protein extracts were quantified by the Bradford method and further examined for the presence of recombinant proteins by 10% sodium dodecyl sulphate–polyacrylamide gel electrophoresis (SDS-PAGE) followed by western blotting using a GFP antibody. A control strain expressing *gpd-gfp* was used as GFP-positive control.

### Analysis of the *rom2Δcnh* Mutant.

One hundred conidia from the *rom2Δcnh* mutant and the parental strain were inoculated onto solid YEPD medium and incubated for 48 h at 37 °C, examined, and photographed. To test the *rom2Δcnh* mutant sensitivity to chemical reagents, serial dilutions of conidia from 10^7^ to 10^4^ were spotted on solid MM (50) containing 50 μg/mL of Calcofluor White, 50 μg/mL Congo Red, 50 μg/mL sodium dodecyl sulphate (SDS), 2.5 μg/mL caspofungin, and 10 μg/mL hygromycin B, respectively. After incubation at 37 °C for 48 h, the plates were photographed. Three independent experiments were performed.

To examine the ultrastructure of the cell wall, mycelia grown in solid MM medium were fixed and examined with an H-600 electron microscope, as described previously (55). For the chemical analysis of the cell wall, conidia were inoculated into 100 mL MM liquid medium and incubated at 37 °C with shaking at 200 rpm for 48 h. The mycelia were harvested, washed with sterile water, and stored at −80 °C. The cell wall components were isolated and assayed as described previously (56). Three samples of lyophilized mycelia were used for cell wall analysis from each strain, and three independent experiments were performed.

### Construction of the *gfp-cnh* Mutant.

Plasmid p434 (51) was used as a backbone construct. A *gfp* and *rom2 cnh* domain (2725-3792) fusion cassette was made and landed with primers P15 and P16 restrictionless into p434 after the *gpd* promoter to induce expression (27) (Table 2). The resulting plasmid *gpd-gfp-rom2* (2725-3792) was transformed into *A. fumigatus KU80ΔpyrG^−^* by PEG-mediated fusion of protoplasts (54). Positive transformants were selected by uridine/uracil autotrophy. The presence of the *gpd-gfp-rom2* (2725-3792) insertions was evaluated by amplifying a 480 bp region of the mutant with PCR primers P17 and P18 (Table 2). Further confirmation in conidia was performed by western blot as described for the *rom2Δcnh* mutant.

### GFP Fluorescence Localization Imaging.

To determine the localization of the GFP-*Af*Rom2, GFP-*Af*Rom2*Δ*CNH, and CNH domain under the *gpdA* promoter, the selected strains (1 × 10^3^ conidia in 1 mL of liquid MM) were grown in eight-well cell culture plates (Thermo Scientific) with a cover glass sitting in the bottom of the well. Live fluorescence imaging was recorded after 13 h incubation at 37 °C with a fluorescence Zeiss Axio Imager M1 equipped with an Axiocam Icc1 and an Axiocam MRM (Carl Zeiss GmbH).

### Protein Extraction and GFP-Trap Affinity Purification.

The *A. fumigatus* strain expressing the *gpd-gfp-cnh* fusion construct and a control strain expressing *gpd-gfp* were grown in YEPD liquid medium with agitation for 24 h at 37 °C. *A. fumigatus* cells were collected by filtering through a Miracloth (Millipore) and dried. Cell extracts were prepared by homogenizing the mycelia, resuspended in lysis buffer (50 mM Tris/HCl pH 7.5, 150 mM NaCl, 50 mM KCl, 0.01% Triton X-100, 1 mM PMSF, and 1:100 protease inhibitor mixture) using liquid nitrogen. Total cell lysates were centrifuged for 30 min at 5,000 rpm to eliminate cell debris before a further centrifugation at 7,000 rpm for 10 min at 4 °C was performed. Total soluble protein concentration was determined by Bradford and then diluted to ∼10 mg/mL before the GFP-Trap affinity purification (Chromotek). GFP-Trap resin (25 μL) was equilibrated in 400 μL of ice-cold dilution buffer (10 mM Tris/HCl pH 7.5, 150 mM NaCl, 0.5 mM EDTA, 1 mM PMSF, 1:100 protease inhibitor mixture) by washing three times according to the manufacturer’s instructions. The GFP-Trap resin was then resuspended in 100 μL of ice-cold dilution buffer, mixed with cell lysate, and incubated for 2 h at 4 °C with gentle agitation. The suspension was centrifuged at 2,000 rpm for 10 min at 4 °C, and the pelleted GFP-Trap beads were washed twice with 500 μL of wash buffer (10 mM Tris/HCl pH 7.5, 350 mM NaCl, 0.5 mM EDTA, 1 mM PMSF, 1:100 protease inhibitor mixture). Bound proteins were extracted in 100 μL glycine pH 2.5 and centrifuged at 2,000 rpm for 2 min at 4 °C. Prior to mass spectrometry, the eluted proteins were separated and analyzed in 10% SDS-PAGE gels.

### Mass Spectrometry and Data Analysis.

Samples from the *gfp-cnh* overexpression mutant and the *gfp* overexpression mutant were run on a 10% SDS-PAGE gel and stained with InstantBlue (Expedeon) Coomassie stain. After thoroughly washing with mass spectometry (MS) grade water (VWR International), each lane on the gel was excised by cutting 1-mm cubes with a sterile scalpel. In-gel digestion and peptides extraction was performed as previously described (57). Each sample (15 μL) was injected for the liquid chromatography–mass spectrometry experiments, which were performed on an Ultimate 3000 RSLCnano System (Dionex- Thermo Scientific) coupled to an LTQ OrbiTrap Velos (Thermo Scientific). The Mascot search engine (Mascot Daemon Version 2.3.2) was used to analyze the data against the *A. fumigatus* proteome (from UniProt, *Neosartorya fumigata* [strain ATCC MYA-4609/Af293/CBS 101355/FGSC A1100] [*A. fumigatus*], December 2019). GO analyses were run using GO Slim Mapper (58) and FungiFun 2.2.8 (59).

### Protein Expression and Purification of *Af*Rom2CNH and *Af*Rho1.

Recombinant His6-Rom2 CNH (862-1194) was cloned into a noncleavable 6His version of the expression vector pGEX6P1 (GE Healthcare) by using restriction enzymes *Bam*HI and *Not*I and primers P23 and P24. Then, the C terminus was truncated using the P20 and P19 primers (Table 2). Recombinant *Af*Rho1 (residues 1 to 181) was cloned into the expression vector pGEX6P1 (GE Healthcare) using restriction enzymes *Bam*HI and *Not*I and primers P21 and P22 (Table 2). A F25N mutation was introduced in *Af*Rho1 for protein stability, as described previously (60, 61). Both expression constructs were transformed into *E. coli* BL21 (non-DE3) cells and plated onto solid Luria Bertani (LB) media supplemented with ampicillin. Positive clones from each construct were inoculated in 100 mL of liquid LB and let them grow overnight at 37 °C in constant agitation. Then, 10 mL of each starter culture was inoculated into 1 L of LB and grown at 37 °C until reaching OD_600_ of 0.8, then protein expression was induced with 250 μM IPTG at 25 °C for 16 h. Induced cultures were harvested by centrifugation at 4,000 rpm at 4 °C for 30 min in a J6-MI centrifuge (Beckman Coulter). Pellets were resuspended in Tris-buffered saline (TBS)) buffer supplemented with 0.5 mM Tris(2-carboxyethyl)phosphine (TCEP) and protease inhibitor mixture (1 mM benzamidine, 0.2 mM PMSF, 5 μM leupeptin) and then lysed by using pressure homogenization with an EmulsiFlex cell disruptor (15,000 kPa; Avestin). This was followed by one step of centrifugation at 16,000 rpm for 1 h using an Avanti J26S centrifuge (Beckman Coulter). Recombinant *Af*Rom2 CNH was enriched by incubating supernatants with His-Nickel beads equilibrated in TBS buffer (GE Healthcare) at 4 °C on a rotating platform for 2 h. Beads were washed five times with TBS to remove nonspecifically binding proteins, and then recombinant *Af*Rom2 CNH protein was eluted by using a 10 to 250 mM imidazole gradient.

Recombinant GST-tagged *Af*Rho1 protein was enriched by using the same procedure described for *Af*Rom2 CNH but using Glutathione-Sepharose beads 4B (GE Healthcare) instead of nickel beads. Recombinant *Af*Rho1 was recovered from the beads by the GST tag cleavage with the addition of 200 μg of PreScission protease at 4 °C overnight in a rotatory shaker.

Eluted proteins were concentrated to 5 mL (*Af*Rom2 CNH) or 2 mL (*Af*Rho1) using a 10 kDa cutoff Vivaspin concentrator (Amersham Bioscience) and further purified by size exclusion using a Superdex 200 column for *Af*Rom2 CNH or a Superdex 75 column for *Af*Rho1 (GE Healthcare). Both columns were previously equilibrated in purification buffer (50 mM Tris, 150 mM NaCl, 0.5 mM TCEP). Proteins were eluted at a flow rate of 1 mL/min in the same buffer using an AKTA prime FPLC system (GE Healthcare). The presence of recombinant protein was confirmed by SDS-PAGE. Fractions containing protein were pooled and concentrated to 2 mg/mL using a 10 kDa cutoff Vivaspin concentrator (GE Healthcare) and then frozen in liquid nitrogen and finally stored at −80 °C until further use.

### Crystallization, Data Collection, and Structure Determination.

*Af*Rom2 CNH and SeMet derivative crystals were grown by vapor diffusion from solutions containing 0.2 μL protein (2 mg/mL) and 0.2 μL of Morpheus H10 condition (Molecular Dimensions) as a precipitant. Crystals were then mounted in nylon loops and flash frozen in liquid nitrogen. Native diffraction data sets were collected at the Diamond Light Source I03 beamline (Harwell), while *Af*Rom2 CNH SeMet derivative datasets were collected at the European Synchrotron Radiation Facility (ESRF) ID30A-3 beamline (ESRF).

*Af*Rom2 CNH and SeMet derivative crystals both belonged to the *P*2_1_ 2_1_2 orthorhombic space group with similar unit cell dimensions. Diffraction data sets were processed using XDS (62) and then merged and scaled with Aimless (63). Experimental phases were obtained from the derivative data by Selenium Single-wavelength anomalous diffraction (Se-SAD) phasing with the AutoSol and Autobuild options in PHENIX program suite (64). This was followed by iterative cycles of manual model building in COOT (65) and structure refinement with REFMAC5 (66). The native *Af*Rom2 CNH structure was solved by molecular replacement using the SeMet *Af*Rom2 CNH model in MOLREP (67, 68). Refmac5 (67) was used for further refinement followed by manual model building with COOT (65). All data collection and refinement statistics are presented in Table 1. Figures depicting the protein structure were generated using PyMOL (69).

*Af*Rho1 crystals were grown by vapor diffusion from solutions containing 0.2 μL protein (20 mg/mL) and 0.2 μL of precipitant consisting of 37.1% wt/vol PEG 5000 MME, 150 mM Tris pH 8.0, and 40 mM magnesium sulfate, plus GDP or GTPγS phosphonucleotides as required. Crystals were mounted in nylon loops and flash frozen in liquid nitrogen, and datasets were collected in the beamline ID30A (ERSF) (GDP) or by using our in-home Rigaku source (GTPγS). Datasets were indexed and integrated using iMosflm (70, 71) and then merged and scaled by Aimless (63). *Af*Rho1 protein complexed with GDP crystallized into the tetragonal space group *P4*_*3*_, while the GTPγS complex crystallized into the *P2*_*1*_*2*_*1*_*2* orthorhombic space group. In both cases, a single molecule was present in the asymmetric unit. *Af*Rho1 structures were solved by molecular replacement using MOLREP (72) with the *Hs*RhoA structure (PDB ID 1FTN) (34) as the phase donor. This was followed by iterative cycles of manual model building in COOT (65) and structure refinement with REFMAC5 (66, 73). Data collection and refinement statistics are presented in Table 1. Figures depicting the protein structures were generated using PyMOL (69).

### BLI Binding Affinity Measurements.

BLI was used to measure the binding affinities of *Af*Rho1 protein with/without GDP/GTPγS nucleotides to *Af*Rom2 CNH. To achieve this, a solution of 1 mg/mL of *Af*Rom2 in reaction buffer (25 mM Hepes, 150 mM NaCl, 0.5 mM TCEP pH 7.5) was biotinylated using the EZLink NHS-Peg4-Biotin reagent (Thermo Scientific) according to the manufacturer’s instructions. Excess of biotin was removed with a 2 mL Zeba spin desalting column (Thermo Scientific). An Octet Htx (Forte Bio) was used for measuring the *Af*Rom2 CNH− apo *Af*Rho1 interaction, and an Octet Red 384 system (Forte Bio) was used for measuring the *Af*Rom2 CNH− *Af*Rho1-GDP and *Af*Rom2 CNH− *Af*Rho1-GTP interactions. Briefly, sensors were coated in each Octet system with the biotinylated *Af*Rom2 CNH and binding experiments performed. The optimal *Af*Rom2 concentration required to coat the super streptavidin (SSA) biosensors, previously soaked in reaction buffer for this experiment, was 30 µg/mL. To block any free SSA streptavidin sites and avoid potential unspecific signals, a further incubation for 120 s with biocytin was required. As a reference control for the experiment, a new set of SSA sensors not exposed to *Af*Rom2 CNH were blocked with biocytin following the same procedure.

The *Af*Rho1–*Af*Rom2 binding consisted of a six-point concentration-dependent series commencing at 1 mM in threefold serial dilutions measured in three steps: 1) 60 s baseline determination in assay buffer, 2) 60 s association in each concentration, and 3) 120 s of dissociation step in assay buffer. The entire experiment was repeated with the control (reference) sensors. Data were processed and visualized using proprietary Octet software specific for each Octet machine, and the response rate was determined by subtracting the baseline and reference responses. Binding isotherms were fit using Octet software to determine the dissociation constant (*K*_d_). The *K*_d_ value was double referenced by applying global, steady-state, and partial fits (where appropriate).

## Supplementary Material

Supplementary File

Supplementary File

Supplementary File

## Data Availability

The atomic models have been deposited in the PDB Database under the accession nos. 5O51, 5ZVP, and 6JIK).
