# Supplementary material for: The citron homology domain as a scaffold for Rho1 signaling
Source: Proc Natl Acad Sci U S A. 2021 Sep 20;118(39):e2110298118. doi: 10.1073/pnas.2110298118 (PMC8488606; doi:10.1073/pnas.2110298118)
Supplement: Supplementary File [file pnas.2110298118.sapp.pdf]

Supplementary Information for:

The citron homology domain as a scaffold for Rho1 signalling

Sergio G. Bartual, Wenfan Wei, Yao Zhou, Wenxia Fang, Kaizhou Yan, Andrew T. Ferenbach, Deborah E. A. Lockhart and Daan M. F. van Aalten

Corresponding autor:

Daan M. F. van Aalten

Email: d.m.f.vanaalten@dundee.ac.uk

This PDF file includes:

Figures S1 to S7
Legends for Figures S1 to S7
Legend for Movie S1
Legend for Dataset S1
SI References

Other supplementary materials for this manuscript include the following:

Movies S1
Datasets S1

S1: Sequence conservation of the Rom2 CNH domain

(A) Sequence alignment of the *Af*Rom2 CNH domain to the CNH domains from human and *Drosophila* citron kinases. Black represents identical and grey represents conserved amino acids. **(B)** Multiple protein sequence alignment of the Rom2 CNH domains from fungal species *Aspergillus fumigatus (Af)*, *Aspergillus nidulans (An)*, *Candida albicans (Ca)*, *Crytococcus neoformans (Cn)*, *Saccharomyces cerevisiae (Sc)*, *Schizosaccharomyces pombe (Sp).* All alignments were performed with ClustalW2.

A

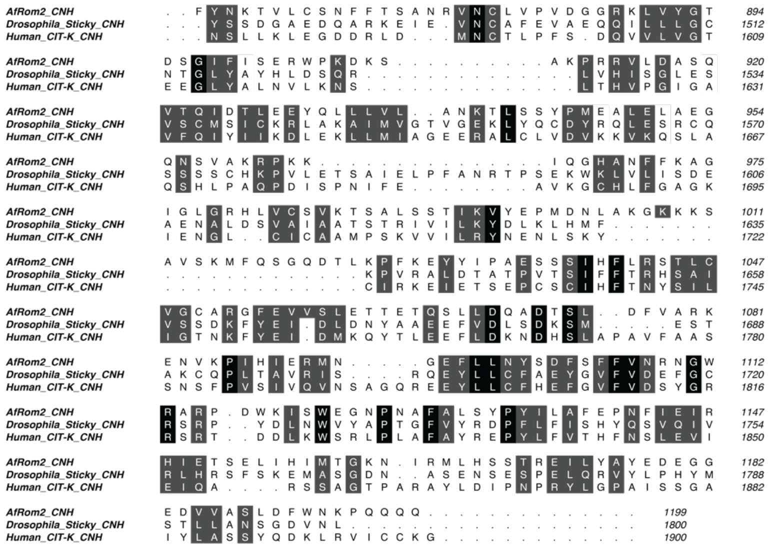

B

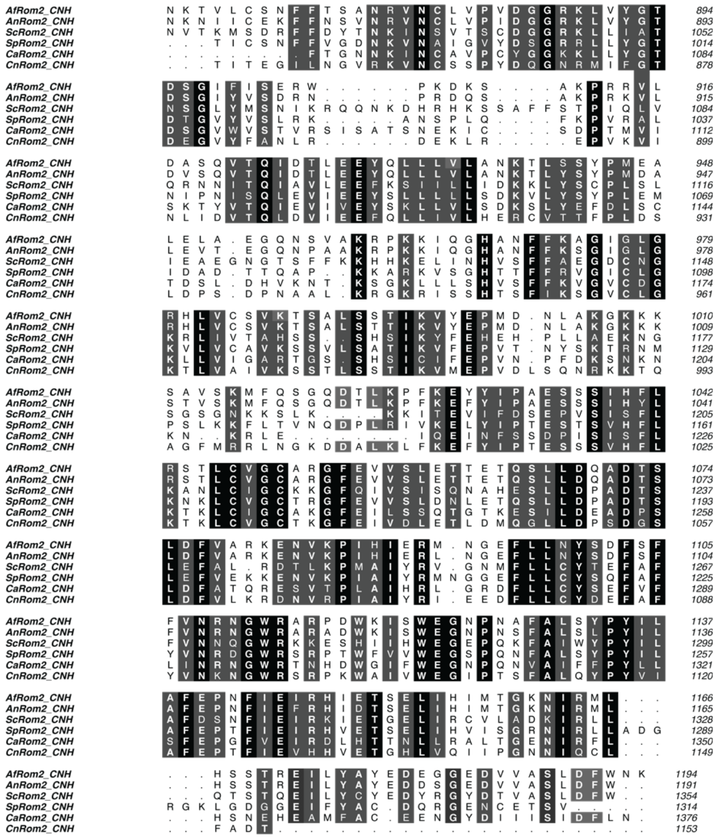

S2. Analysis of the parental and *rom2Δcnh* mutant localisation and cell wall defects. (A) GFP fluorescence images (GFP) showing the cellular localisation of the *Af*Rom2-GFP, the *Af*Rom2ΔCNH-GFP and the *Af*CNH-GFP proteins. The Bright Field (BF) and the merge is provided for clarity. (B) TEM images of representative parental hyphal cells. (C) TEM images of representative *rom2Δcnh* mutant hyphal cells.

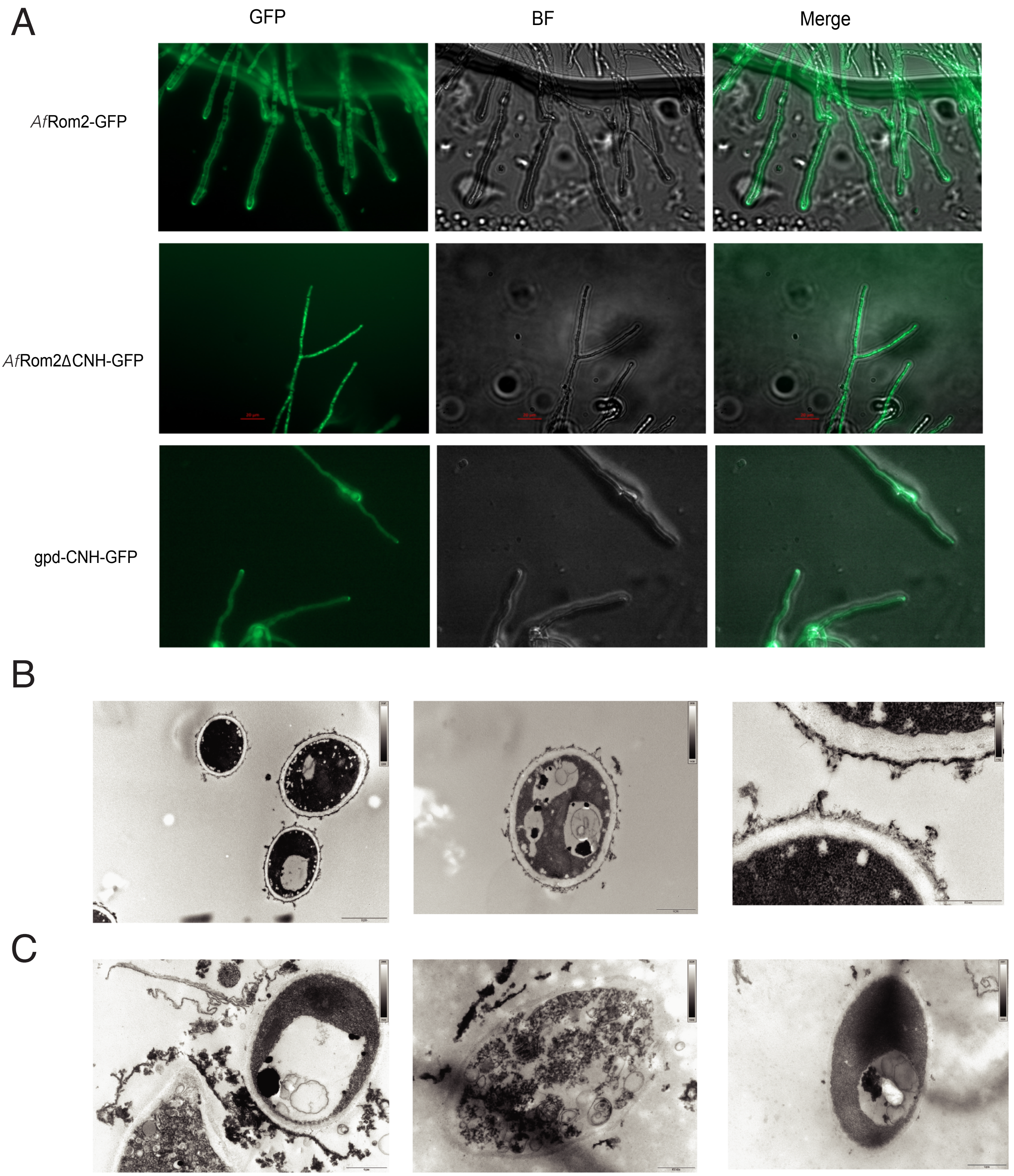

S3: Generation of *gfp-cnh* mutant, pull down experiments and binding affinities.

(A) Schematic diagram of construction of the *gfp-cnh* mutant. The *gfp-cnh* fusion cassette was randomly integrated into the *A. fumigatus* genome. **(B)** PCR confirmation of two transformants as the *gfp-cnh* mutants. Two mutants (Mt1 and Mt2) produced the correct size of the band (420 bp), **(C)** Western Blot using the *gfp-cnh* mutant cell lysate and GFP antibody, with a *gfp* control strain as a positive control, and the parental strain as negative control. The size difference between the Mt1 and Mt2 to the *gfp* control strain represents the correct molecular weight of the CNH protein (40 kDa). **(D)** 10% SDS-PAGE gel of the GFP-CNH pull down, a mutant strain expressing GFP protein alone was used as a control strain. Input lanes are cell lysate after the cell lysis, FT (flow through) lanes are protein after the GFP beads binding, elution lanes are proteins eluted from the GFP beads. **(E)** Cellular localisation analyses of proteins identified by GFP-CNH pull down MS.

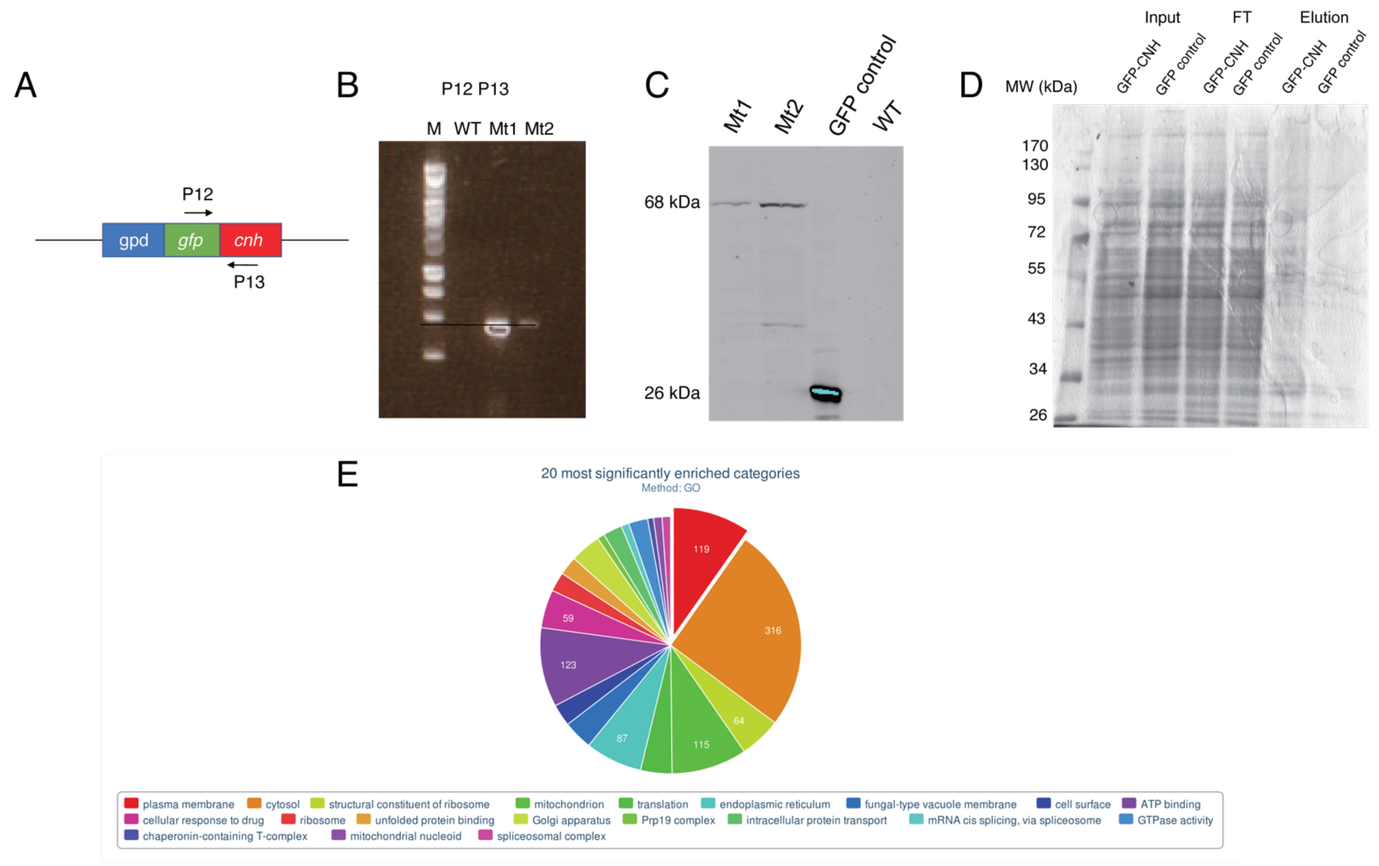

S4: BLI response curves obtained for the apo *Af*Rho1, *Af*Rho1 in complex with GDP or in complex with GTPγS against immobilized *Af*Rom2. Thermodynamic dissociation constant calculations are shown on top of each response curve.

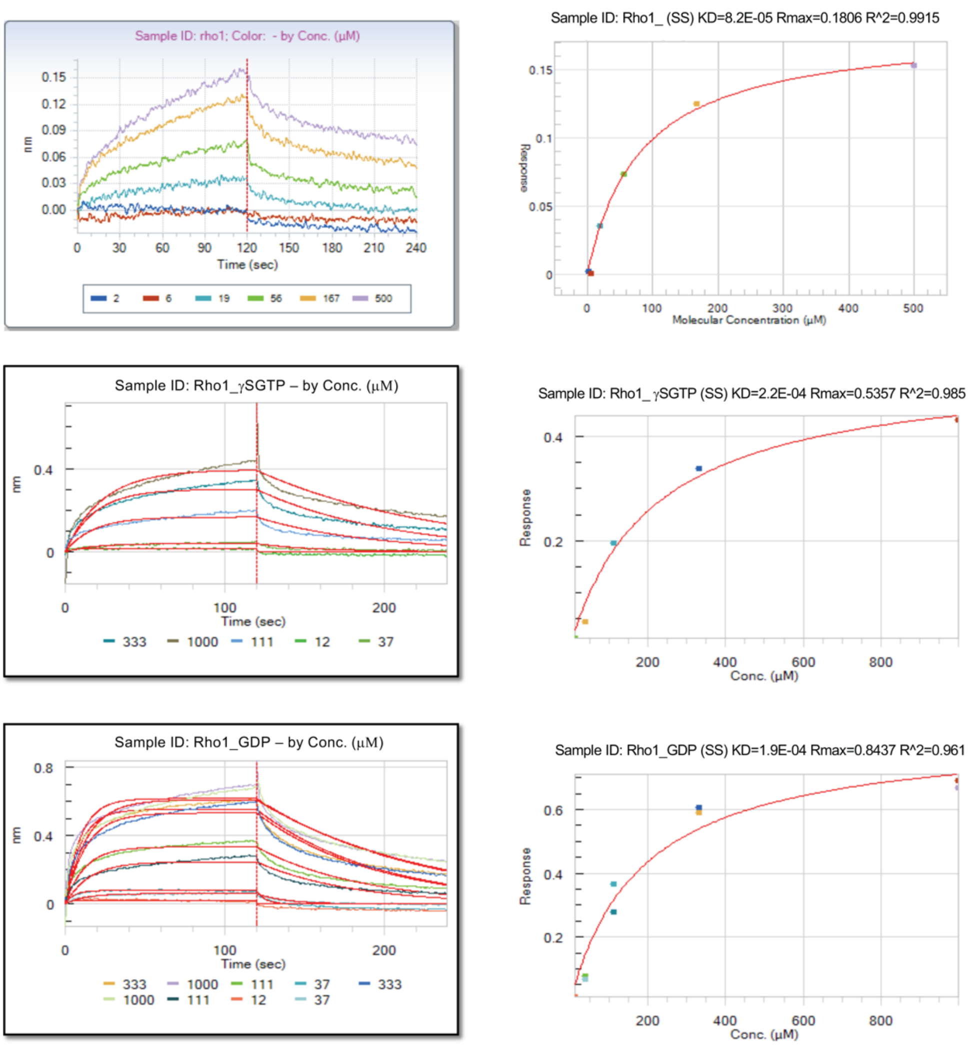

S5: Analysis of the *Af*Rom2 CNH domain structure.

(A) Structure-based sequence alignment of the CNH domain with similar β-propeller structures performed with the Dali server (1). Cross section diameters of the AfRom2 ellipsoid are shown as dashed arrows and values in Å are indicated. The inset contains the detailed sequence alignment of the CNH domain L2,L3 loops with CNH domains from *Saccharomyces* Rom2 (*Sc*Rom2), *Drosophila* Sticky (*Dm*_Sticky) and human citron kinase (*Hs*_CIT_K). **(B)** Stereo view of the *Af*Rom2 CNH structure highlights the inter-blade contacts.

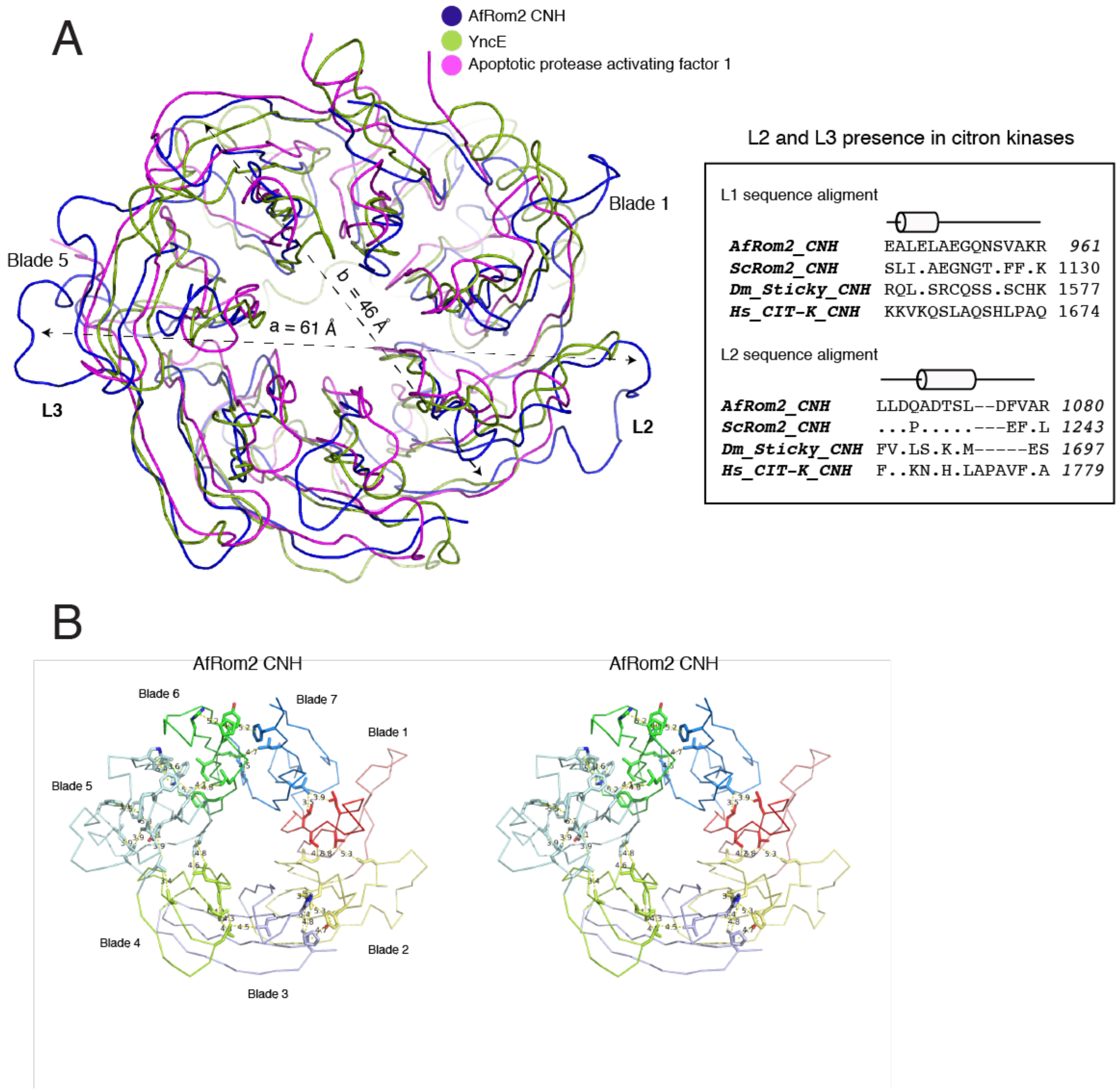

S6. Crystal structure of *Af*Rho1 in complex with GDP. (A) Electron density of GDP (left) and GTP-γ-S (right) with Mg^{2+} and interactions with *Af*Rho1. Unbiased F_o-F_c maps are contoured at 1.5 σ. **(B)** Superposed structures of *Af*Rho1 (light blue) and its human orthologue *Hs*RhoA (wheat). GDP and magnesium are shown as sticks and spheres respectively. **(C)** Sequence alignment of *Af*Rho1 and Hs*RhoA* indicating the main structural motifs. The black arrow indicates the stabilising mutation discussed in the Materials and Methods.

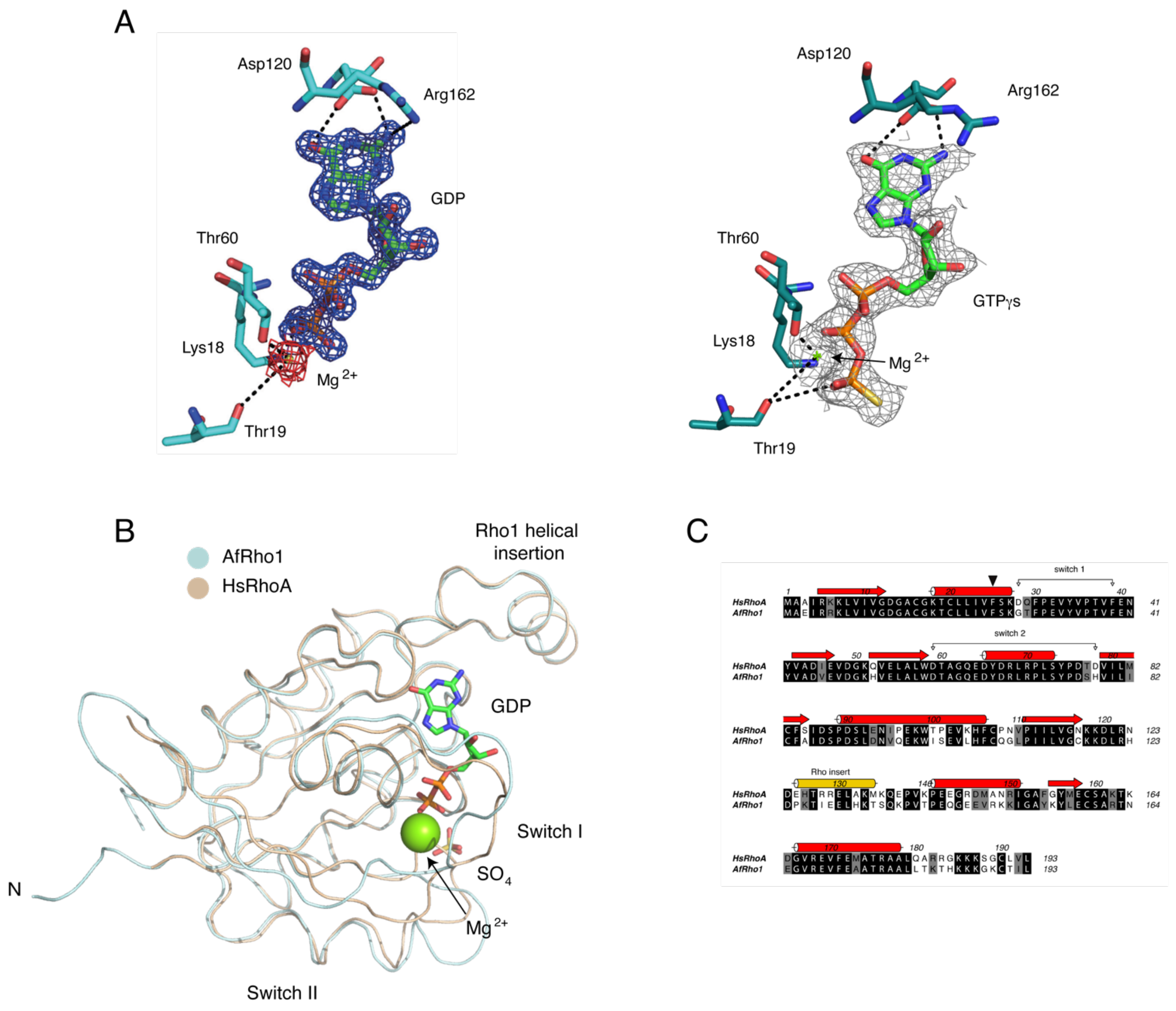

S7. Analysis of the *Af*Rho1-*Af*Rom2 CNH domain complex model. The homology model of the *Af*Rho1 - *Af*Rom2 CNH domain complex described in the main text is compared to the crystal structure of the Nidogen – Laminin protein-protein complex (PDB 1NPE). The *Af*Rho1 Switch II helix is coloured red, while the Laminin loop interacting with Nidogen is coloured orange. In both models, the interacting residues are labelled and shown as sticks for clarity.

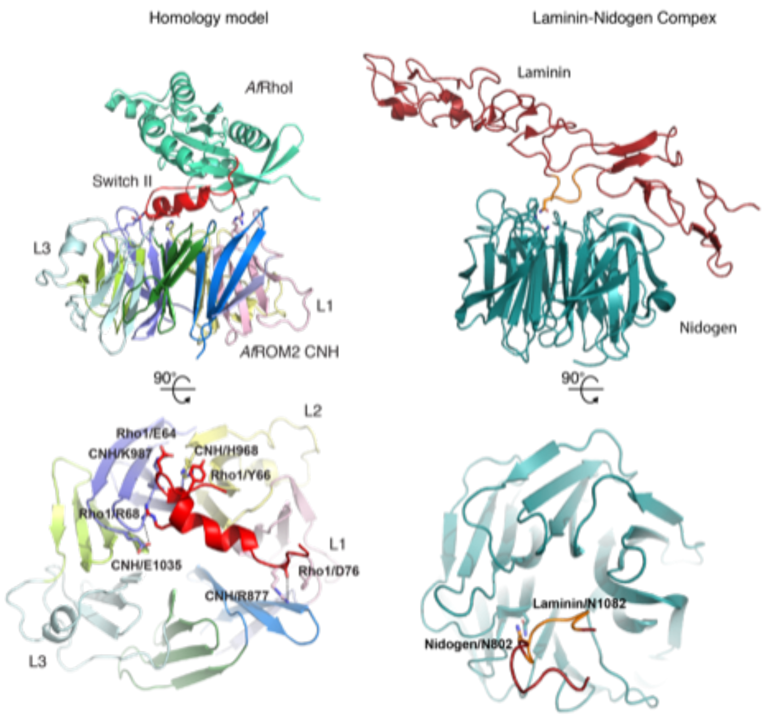

Video S1. Conformational changes between the active Rho1 in complex with GTPγS and the relax Rho1 in complex with GDP. Switch I motif is coloured in magenta while Switch II is coloured in red. For clarity, GTPγS and GDP have been removed from the structures**.**

Dataset S1. List of CNH pull down interactors obtained by mass spectrometry analysis.

SI References

1. L. Holm, L. M. Laakso, Dali server update. *Nucleic Acids Res* **44**, W351-355 (2016).
2. R. J. Redkar, R. W. Herzog, N. K. Singh, Transcriptional activation of the Aspergillus nidulans gpdA promoter by osmotic signals. *Appl Environ Microbiol* **64**, 2229-2231 (1998).
